# Intra-Rater (Live vs. Video Assessment) and Inter-Rater (Expert vs. Novice) Reliability of the Test of Gross Motor Development—Third Edition

**DOI:** 10.3390/ijerph18041652

**Published:** 2021-02-09

**Authors:** Aida Carballo-Fazanes, Ezequiel Rey, Nadia C. Valentini, José E. Rodríguez-Fernández, Cristina Varela-Casal, Javier Rico-Díaz, Roberto Barcala-Furelos, Cristian Abelairas-Gómez

**Affiliations:** 1 Health Research Institute of Santiago de Compostela (IDIS), University Hospital of Santiago de Compostela-CHUS, 15706 Santiago de Compostela, Spain; aidacarballofaz@gmail.com (A.C.-F.); roberto.barcala@uvigo.es (R.B.-F.); 2 CLINURSID Research Group, Psychiatry, Radiology, Public Health, Nursing and Medicine Department, Universidade de Santiago de Compostela, 15782 Santiago de Compostela, Spain; 3 REMOSS Research Group, Faculty of Education and Sport Sciences, Universidade de Vigo, 36005 Pontevedra, Spain; zequirey@uvigo.es (E.R.); cristinavarelacasal@uvigo.es (C.V.-C.); 4 Department of Physical Education, Physical Therapy and Dance, Universidade Federal do Rio Grande do Sul, Porto Alegre 90690-200, Brazil; nadiacv@esef.ufrgs.br; 5 Faculty of Education Sciences, Universidade de Santiago de Compostela, 15782 Santiago de Compostela, Spain; geno.rodriguez@usc.es (J.E.R.-F.); javier.rico.diaz@usc.es (J.R.-D.)

**Keywords:** TGMD-3, agreement, assessment, fundamental motor skills, raters, live, slow-motion

## Abstract

The Test of Gross Motor Development (TGMD) is one of the most common tools for assessing the fundamental movement skills (FMS) in children between 3 and 10 years. This study aimed to examine the intra-rater and inter-rater reliability of the TGMD—3rd Edition (TGMD-3) between expert and novice raters using live and video assessment. Five raters [2 experts and 3 novices (one of them BSc in Physical Education and Sport Science)] assessed and scored the performance of the TGMD-3 of 25 healthy children [Female: 60%; mean (standard deviation) age 9.16 (1.31)]. Schoolchildren were attending at one public elementary school during the academic year 2019–2020 from Santiago de Compostela (Spain). Raters scored each children performance through two viewing moods (live and slow-motion). The ICC (Intraclass Correlation Coefficient) was used to determine the agreement between raters. Our results showed moderate-to-excellent intra-rater reliability for overall score and locomotor and ball skills subscales; moderate-to-good inter-rater reliability for overall and ball skills; and poor-to-good for locomotor subscale. Higher intra-rater reliability was achieved by the expert raters and novice rater with physical education background compared to novice raters. However, the inter-rater reliability was more variable in all the raters regardless of their experience or background. No significant differences in reliability were found when comparing live and video assessments. For clinical practice, it would be recommended that raters reach an agreement before the assessment to avoid subjective interpretations that might distort the results.

## 1. Introduction

Fundamental movements skills (FMS) consist of a basic organized movement involving the combination of movement patterns of two or more parts of the body [1]. FMS are considered “building blocks” for more advanced and complex movements necessary to participate in different sports, games and other physical activities. Commonly, FMS are classified into locomotor skills (e.g., run, jump, hop, slide), object control/ball skills (e.g., catch, kick, strike, throw) and balance/stability skills (e.g., static balance, dynamic balance) [1,2,3].

Strong existing evidence suggests positive associations between FMS competency and physical activity [4], physical fitness [4,5] and health-related benefits [6,7] such as healthy weight status [4,5], cardiorespiratory fitness [4,5] or positive cognitive and academic outcomes [8] among others. However, the acquisition of FMS proficiency levels does not occur naturally and the implementation of structured physical education programs in pre-school and school-aged children is required [9].

In light of the aforementioned reported benefits, existing literature supports the importance of assessing FMS using valid and reliable tools [10], which is also necessary to provide valuable information about children’s motor performance and progress [11]. It is essential to early identification of possible delays or disorders that could affect motor competence and cognitive [12] and affective [13] development.

There are several assessment tools to evaluate FMS, which can be broadly classified into two categories: quantity/product-oriented assessment, which evaluates the outcome of the movement (e.g., velocity, trajectory) and quality/process-oriented assessment, which evaluates the pattern of the movement (e.g., how a person throws) [14,15]. Moreover, assessments may examine gross motor skills, movements that require the use of large motor groups (e.g., running, jumping) [16] or fine motor skills, movements that involve small motor groups (writing, eating) [17].

One of the most common assessments used to examine FMS in children is the Test of Gross Motor Development (TGMD) [3] and its variants TGMD—Second edition (TGMD-2) [18] and TGMD—Third Edition (TGMD-3) [19]. The TGMD-3 is a validated and reliable [20] process-oriented assessment applied to evaluate gross motor competence in children between 3 years-0 months and 10 years-11 months. The TGMD-3 assess thirteen FMS divide into two subscales, locomotor and ball skills.

Reliability is one of the most essential and fundamental features in assessing performance in research. For one hand, reliability is an essential issue in research, since it lets researchers replicate studies. On the other hand, from a practical perspective, it would be important to assess accurately those variables that wanted to be studied. For example, regarding the scope of the present study, the evaluation of FMS by schoolteachers or clinical staff. In this sense, a current systematic review [20] of 23 studies assessing the reliability of the TGMD showed good-to-excellent inter-rater reliability, good-to-excellent intra-rater reliability and moderate-to-excellent test-retest reliability. Most studies assessed the reliability among experienced raters using video evaluation. However, the TGMD is sometimes used by researchers or practitioners (such as physical education teachers) with little or no training [21], since the examiner’s manual only recommend to train with at least three children before a diagnostic evaluation [18,19]. Thus, it is necessary to know reliability among raters with different backgrounds and experiences. Only one previous study has compared differences in scores between expert and novice coders [22]. The results indicated that novice raters could not score the TGMD-2 in a significantly similar manner to the experts [22]. However, to this day, no study examined this issue in TGMD-3. 

Although the TGMD examiner’s manual does not refer to the children’s assessment through video recordings, many studies used videotaped assessment because of its advantages over live evaluation. It allows the evaluation of each skill’s criteria in more detail and repeatedly, even in slow-motion if necessary [22]. Intra and inter-rater reliability between expert and novice raters considering a different type of video viewing, live and slow-motion, has not yet been investigated. Thus, the purpose of this study was to analyze intra- and inter-rater reliability of TGMD-3 using live and video assessments between experts and novice raters.

## 2. Materials and Methods

### 2.1. Participants

This study was conducted with 25 healthy children (60% females, *n* = 15; 100% Hispanic, *n* = 25) between 8 and 10 years old (Mean ± SD: 9.16 ± SD 1.31 years), attending a public elementary school during the academic year 2019-2020 in Santiago de Compostela, Galicia, Spain. Participants’ anthropometric were obtained for each child (height: 1.37 ± 0.11 m; weight: 34.57 ± 8.49 kg; and body mass index: 18.26 ± 2.95 kg·m^−2^). 

Participation in the study was voluntary and previously, parents or guardians signed the informed consent. All children were provided with verbal information and gave verbal assent before the test. This study respected the Helsinki Convention’s ethical principles and was approved by the Ethical Committee of the University of Vigo, Spain.

### 2.2. Raters

Five raters (convenience sample), two experts (ER) and three novices (NR) were responsible for assessing the participants’ video recorders. The three NR had no previous experience rating TGMD-3. One of the novices had a BSc in Physical Education and Sports Science (NR-PE); the other two were a primary schoolteacher and a nurse, both with no physical education or sports science background and experience in working with children either. However, all of them might have to assess FMS of children in the future due to their academic/professional background. Before coding children’s performance, NR studied the content of the TGMD manual [19], enquired the expert coders about their doubts and practiced administering the TGMD-3 to three children. The two ER were BSc in Physical Education and Sports Science. Both with more than 5 years of experience testing the TGMD.

### 2.3. Test of Gross Motor Development—Third Edition [TGMD-3]

The TGMD-3 assesses gross motor skill performance in children between 3 years and 0 months and 11 years and 11 months. It consists of two subscales: locomotor and ball skills. The locomotor subscale measures the gross motor skills that involve fluid coordination of the body while the child moves in one direction or another and includes: run, gallop, hop, skip, horizontal jump and slide. The ball skills subscale assesses the gross motor skills that require effectiveness in intercepting and propelling objects and involves: striking a stationary ball, forehand strike, stationary dribble, catch, kick, overhand throw and underhand throw. Each skill, which includes several behavioral components, was evaluated through three to five criteria, scored 1 or 0 depending on the presence or absence [19]. We obtain independent scores for each skill, subscale and total test from the criteria scores.

After a verbal description and a practical demonstration, the children perform three trials of each skill. The first trial is a practice and it was not scored. The examiner scores the remaining two trials as follows: if the child performs a behavioral component or criterion correctly, the examiner scores a 1 and if the child does not perform it correctly, a 0. According to this, the maximum score that each child can achieve is 100 points (46 for the locomotor subscale and 54 for the ball skills subscale).

### 2.4. Procedures

A physical education teacher and a nurse (PhD student) conducted the assessments. All data were collected in March 2020. The tests were carried out in a sports hall, during the school’s regular schedule and always in the presence of at least one teacher at the school. The testers provided the children with a verbal description of each skill, followed by a video showing the skill’s correct performance (showed twice: normal speed and slow-motion) following the TGMD-3 guidelines [19]. Each child performed a practice trial followed by two consecutive trials, which were video-recorded (camera Nikon D5300) for later reliability analysis. The administration of the test to each child took approximately 20–30 min.

The five raters independently assessed the recorded videos to analyze the inter- and intra-rater reliability. Raters assessed the video in two viewing modes: live (once, at normal speed and without pause) and slow-motion (watching the video as many times as they needed, in slow-motion and with pauses). The children, the order of skills and the type of visualization (live/slow-motion) for each video was randomized for each rater. For the study of intra-rater reliability, each rater re-assessed the videos following the corresponding viewing mode according to the randomization after 2 weeks. In this sense, intra-rater reliability was analyzed according to the viewing mode (live vs. slow-motion) and the inter-rater reliability was studied between NR and ER in both conditions, live and slow-motion.

### 2.5. Statistical Analysis

Reliability was assessed by intraclass correlation coefficients (ICC) for both intra- and inter-rater. The selection process of ICC was based on Koo et al. [23] flow-chart. ICC values and their 95% confident intervals for intra-rater reliability were based on single-measurement, absolute-agreement, 2-way mixed-effects model. In the case of inter-rater reliability, single-measurement, consistency, 2-way random-effects model. Values less than 0.5 indicate poor reliability, values between 0.5 and 0.75 indicate moderate reliability, values between 0.75 and 0.9 indicate good reliability and values greater than 0.90 indicate excellent reliability.

Additionally, repeated-measures ANOVA was performed to analyze the overall scores and the locomotor and ball skills subscales’ scores; means and standard deviations were provided. Two factors were integrated into the model: one intra-group (live vs. slow-motion) and one inter-group (raters). Raters were categorized as follows: NR (Rater A and Rater B), NR-PE (Rater C) and ER (Rater D and Rater E).

All analyses were performed using the SPSS statistical package version 23 (SPSS Inc, Chicago, IL). A significance level of *p* < 0.05 was considered.

## 3. Results

### 3.1. Intra-Rater Reliability: Live vs. Slow-Motion

Results of intra-rater reliability are shown in Table 1. Good-to-excellent or excellent reliability were found for most of the skills, mainly in ER and NR-PE evaluations. ICC values from subscales’ and overall scores also showed good-to-excellent or excellent reliability in the case of ER and NR-PE. NR showed moderate-to-excellent, good-to-excellent or excellent intra-rater reliability in both subscales’ and overall scores.

ER and NR-PE achieved intra-rater reliability at least moderate in all skills (except ER (Rater E): slide). In the case of NR, run (Rater A and B), hop (Rater B), horizontal jump (Rater A and B), kick (Rater A) and underhand throw (Rater B) were the skills in which reliability was poor-to-moderate/good.

Descriptive statistics of overall score and locomotor and ball skills subscales’ scores are presented in Table 2. Significant differences in the test factor (live vs. slow-motion) were found in locomotor subscale in repeated measures analysis (live: 33.7 ± 4.0; slow-motion: 33.2 ± 3.9; *p* = 0.010). The post-hoc Bonferroni analysis revealed significant differences between live and slow-motion evaluations in both NR (Rater A: *p* = 0.014/Rater B: *p* = 0.036) in the locomotor subscale. Same differences were found in the ball subscale in one of the NR (Rater B: *p* = 0.001) and overall score in the same NR (Rater B: *p* < 0.001) and one ER (Rater D: *p* = 0.038).

### 3.2. Inter-Rater Reliability: Novice vs. Experts

**Figure 1:**
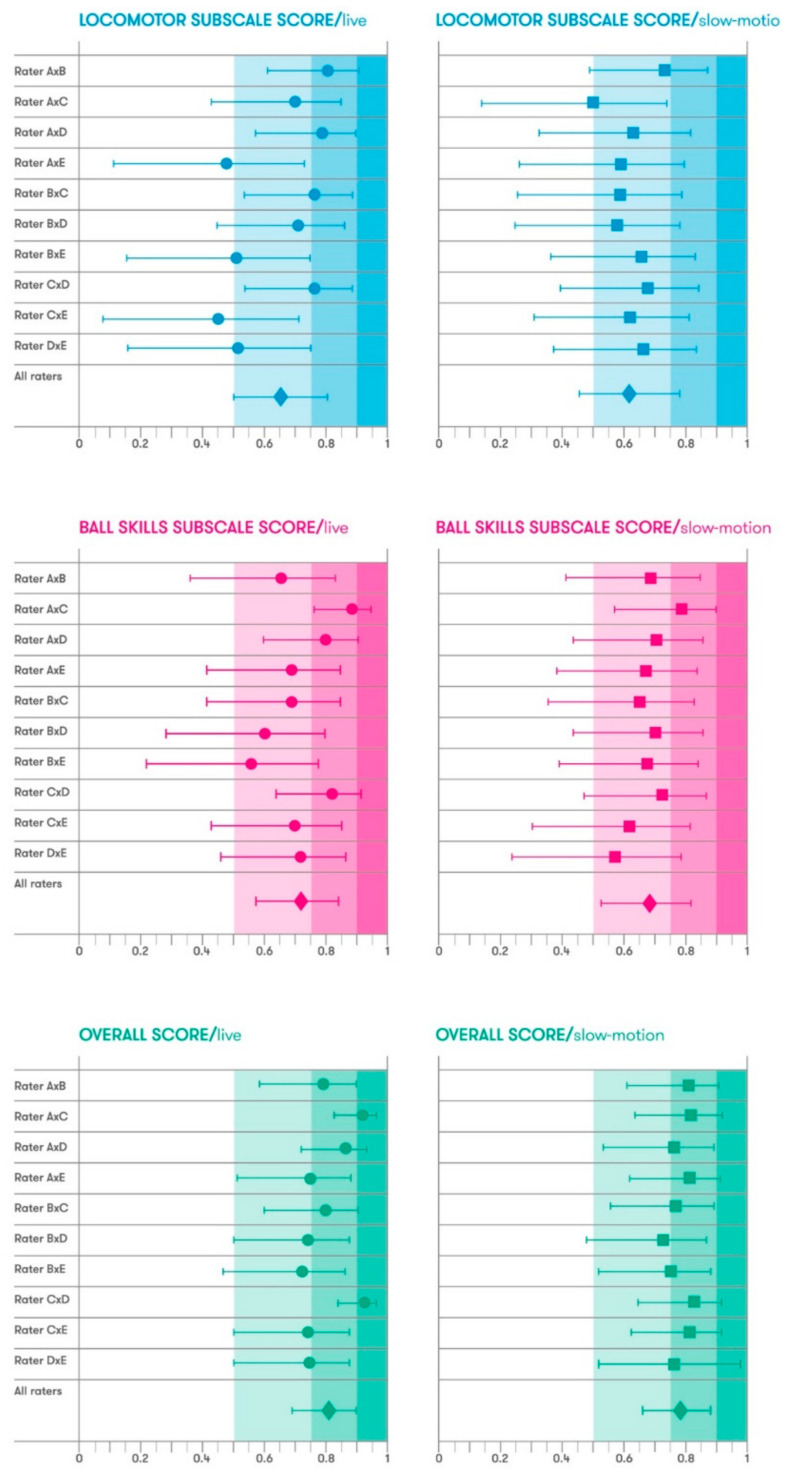
Inter-rater intraclass correlation coefficients (ICC) values of locomotor and ball skills subscales and overall score.

In general, poor-to-moderate inter-rater reliability was found in both locomotor and ball skills, with null changes comparing live vs. slow-motion between each pair of raters. Table 3 and Table 4 present inter-rater ICC results for each skill. Figure 1 presents the ICC associations with locomotor and ball subscales and overall scores.

Regarding locomotor skills, reliability were moderate-to-good; for most cases, independent of the rater’s experience or background. Gallop was the skill with the best inter-rater reliability; horizontal jump and skip reached ICC values over 0.5 in most comparisons. Hop was the locomotor skill with lower inter-rater reliability.

Regarding ball skills, the two-hand strike was the skill with higher inter-rater reliability, ranging between poor-to-good and moderate-to-excellent. The two-hand catch was the skill with the lower inter-rater reliability.

Ball skills subscale and overall scores are slightly higher in ER compared with NR and NR-PE (Table 2). The repeated measures analysis showed no differences in the interactions for both viewing modes and raters factors for the locomotor subscale. However, regarding ball skills, one of the experts, rater (Rater D), coded significantly higher in live evaluation than one NR (Rater B: *p* = 0.024) and NR-PE (*p* = 0.001). Same differences were found in slow-motion evaluation between Rater D and both NR (Rater A: *p* = 0.029/Rater B: *p* = < 0.001) and NR-PE (*p* = 0.001). Finally, overall scores registered only significant differences between one ER (Rater D) and NR-PE in slow-motion evaluation (*p* = 0.031).

## 4. Discussion

The purpose of this study was to analyze the intra- (live and slow-motion) and inter-rater [expert and novice (one of them with physical education background)] of TGMD-3. Briefly, locomotor and ball skills subscales and overall scores showed moderate-to-excellent intra-rater reliability. The inter-rater reliability showed moderate-to-good scores for the ball skills subscale and overall scores for both live and slow-motion and poor-to-good reliability for the locomotor subscale also for both assessing modes.

The overall score for intra-rater reliability was, at least, moderate-to-excellent. However, ER and NR-PE reached higher levels of reliability than NR, with excellent intra-rater ICC values. In this sense, previous studies also showed good-to-excellent or excellent intra-rater reliability in the TGMD-2 [24,25,26,27] and the TGMD-3 [28,29,30,31,32]. For locomotor and ball skills subscales’ scores, although intra-rater reliability of NR was at least moderate-to-excellent, ER and NR-PE achieved no less than good-to-excellent. High agreement was also reported in both subscales in the TGMD-2 [24,25,26,27] and the TGMD-3 [28,30,31,32,33].

In the stringent analysis performed by skill, all the raters achieved good-to-excellent or excellent intra-rater reliability for gallop, two-hand strike and two-hand catch. Nevertheless, it was observed more differences between raters in intra-rater the individual skills, ICC values compared to overall score or subscales scores. Previous studies also found variations in the intra-rater reliability agreement across raters [31], especially when skills are examined individually [10], obtaining results ranging from poor-to-excellent. In the present study, intra-rater reliability varied from moderate-to-excellent to excellent for ER and NR-PE in all skills (except Slide for Rater E). Variability in ICC values was higher in NR, with moderate-to-good or moderate-to-excellent intra-rater reliability in half of the skills.

Regarding inter-rater reliability, our results show that the degree of consistency varies considerably between raters on the TGMD-3 skills. As observed, the variations in inter-rater reliability were more visible in the analysis by skill. The ICC’s results for the overall test, locomotor and ball skills subscales’ scores were over 0.5 in almost all comparisons. The inter-rater reliability for locomotor skills, among five raters, was poor-to-moderate for run, slide and hop (both live and slow-motion); and poor-to-good for the skip in slow-motion and the horizontal jump (both live and slow-motion); and moderate-to-good for the skip in live viewing and the gallop in both viewing modes. Overall, the skill with the higher reliability among raters was the gallop achieving an ICC > 0.5 in all comparisons, reaching moderate reliability in 15/20 comparisons between raters. In contrast, the lower results of reliability were found for the hop and run skill. Kim et al. [34] have also reported the gallop as the skill with higher reliability results and the run skill as the one with the lowest reliability coefficients. In contrast, Maeng et al. [31] and Ayán et al. [35] achieved the most robust reliability in the hop and the weakest in the gallop, contrary to our findings.

Regarding the ball skills, the inter-rater reliability was poor-to-moderate for one-hand stationary dribble (live), overhand throw (slow-motion), kick, forehand strike, two-hand catch and underhand throw (both live and-slow motion); poor-to-good for two-hand strike (slow-motion), one-hand stationary dribble (slow-motion) and overhand throw (live); and moderate-to-good for the two-hand strike in live viewing. In general, the two-hand strike was the skill with the best reliability among raters reaching an ICC > 0.5 and moderate reliability in 19/20 comparisons. The lowest reliability coefficients achieved were in two-hand catch skill. This result is in concordance with previous studies [36] but again, contrary to others that found the two-hand catch as the skill with stronger inter-rater reliability [10].

Our results show a lower agreement on the locomotor skills compared to the ball skills. Rintala et al. [10] and Palmer and Brian [22], who reported similar results in TGMD-2, suggested that before using TGMD, further training with the locomotor skills may be needed, our results support this contention. On the other hand, it seems that the raters’ background and experience did not influence the inter-rater reliability, taking into account that inter-rater reliability varied not only among NR and ER raters but also between NR each other and ER. Contrary, Palmer and Brian [22] found that novice raters could not achieve a significant agreement with expert raters assessing the TGMD-2 test. However, it has been shown that novice raters reached a significantly similar agreement to experts assessing measure movements with the Landing Error Scoring System [37] and Functional Movement Screen [38].

The variation found in inter-rater reliability in our study could be due to different interpretations of each skill evaluation criteria. Some of them give rise to subjectivity and vary according to the concrete interpretation of the raters. For example, in the hop skill, the second criterion, “Foot of non-hopping leg remains behind hopping leg” might be interpreted differently; it might consider scoring 1 if non-hopping foot always remained behind hopping leg. Nevertheless, it might be considered scoring 1 if the child, although not always, executed the criterion correctly most of the time. Hence, it becomes necessary to obtain pre-assessment agreements between raters or professionals who pretend to evaluate FMS in children. It has been suggested that more training may be necessary for those skills more difficult to assess and even that some of the criteria might be re-defined to make them more objective [10,20,31,34,36]; our results provide further support for this recommendation.

Besides, a recent systematic review about TGMD-2 and 3 reliability showed at least good-to-excellent ICC values for inter-rater reliability in both subscales and the overall score, higher than those obtained in this study. However, these differences might be regarding the methodology adopted or the statistical analysis used [20]. In the case of ICC, used in many studies, several aspects make it challenging to compare the results. First, investigators have to choose an ICC model (One-Way Random-Effects Model, Two-Way Random-Effects Model or Two-Way Mixed-Effects Model), ICC definition (absolute agreement or consistency) and ICC type (single or mean measurement) according to the study. Second, there are no standards values to report reliability. Third, some studies did not report ICC model/type/definition or reported reliability based on ICC values with no mention of the confidence interval. Our study followed the flowchart proposed by Koo et al. [23] for selecting model, type and definition. Many studies have used different models, in most cases, less demanding, which might explain the higher reliability results obtained compared to our study.

About to ICC values and 95% confidence intervals, interpretation was also based on Koo et al. [23] recommendations. In this respect, ICC values do not describe reliability in itself but it is necessary to express reliability in terms of lower and upper bounds of the confidence interval. Finally, we considered excellent reliability if an ICC equal to or higher than 0.9 was obtained, while other ratings considered it above 0.75 [39,40]. All this makes it difficult to compare the reliability used in different studies since this variability causes an interpretation problem.

### Limitations

This study is not free of limitations. Our reliability study was carried out assessing 25 healthy children. The sample was small, so the results should be interpreted with caution. Besides, we tried to simulate the live assessment by displaying the digital records only once and playing the video at a normal speed. This procedure does not represent precisely the reality of a live assessment by professionals who have to score the children’s performance. However, from a research point of view, it is a useful way for different evaluators to carry out the evaluation at any time.

## 5. Conclusions

The TGMD-3 battery showed moderate-to-excellent intra-rater reliability for overall score, locomotor and ball skills subscales’ scores and moderate-to-good inter-rater reliability for overall and ball skills subscale scores and poor-to-good for locomotor subscale. The expert raters and the novice rater with physical education background achieved stronger intra-rater reliability than novice raters and inter-rater reliability did not seem to be influenced by the raters’ background and experience. The viewing modes also seemed not to influence on reliability but further investigation is needed in this regard, in the sight of the study limitations. Higher variability in both intra- and inter-rater reliability was found when analyzing the skills separately.

In conclusion, our high results in terms of intra-rater reliability but lower in the case of inter-rater, suggest that each skill’s criteria can be interpreted differently. For clinical practice, it would be recommended that raters reach an agreement before the assessment to avoid subjective interpretations that might distort the results. In addition, a revision of some criteria might be needed to let research replication.

## Figures and Tables

**Table 1:** Intra-rater reliability *: live vs. slow-motion: Subscales, skills and overall scores.

	NR		NR-PE	ER		Good-to-Excellent or Excellent
	Rater A	Rater B	Rater C	Rater D	Rater E	
Locomotor skills						Number of raters
Run	0.304 (−0.409–0.676)	0.660 (0.236–0.850)	0.900 (0.773–0.956)	**0.913 (0.805–0.961)**	0.942 (0.869–0.975)	3/5
Slide	0.808 (0.567–0.915)	0.921 (0.822–0.965)	**0.966 (0.924–0.985)**	**----- †**	0.714 (0.365–0.873)	2/4
Gallop	0.950 (0.888–0.978)	0.925 (0.823–0.968)	**0.966 (0.923–0.985)**	**0.986 (0.969–0.994)**	**0.976 (0.944–0.989)**	5/5
Hop	0.825 (0.610–0.922)	0.705 (0.332–0.870)	0.936 (0.855–0.972)	**0.990 (0.977–0.996)**	0.884 (0.736–0.949)	2/5
Horizontal jump	0.765 (0.460–0.897)	0.680 (0.166–0.868)	**0.956 (0.900–0.981)**	**0.992 (0.982–0.997)**	0.916 (0.811–0.963)	3/5
Skip	0.892 (0.758–0.952)	0.861 (0.685–0.939)	**1.000**	**0.989 (0.976–0.995)**	**0.958 (0.905–0.981)**	4/5
Locomotor subscale score	0.865 (0.654–0.943)	0.817 (0.592–0.919)	**0.964 (0.918–0.984)**	**0.980 (0.954–0.991)**	0.901 (0.773–0.957)	3/5
**Ball skills**						
Two-hand strike	**0.959 (0.909–0.982)**	0.905 (0.783–0.958)	0.945 (0.876–0.976)	**0.968 (0.925–0.986)**	0.945 (0.876–0.976)	5/5
One-hand stationary dribble	0.917 (0.813–0.964)	0.871 (0.662–0.947)	0.917 (0.803–0.964)	0.883 (0.739–0.948)	0.910 (0.798–0.960)	3/5
Overhand throw	0.931 (0.844–0.970)	0.887 (0.742–0.950)	0.934 (0.852–0.971)	**0.975 (0.942–0.989)**	0.915 (0.807–0.963)	4/5
Kick	0.659 (0.230–0.849)	0.846 (0.625–0.934)	0.839 (0.537–0.936)	0.948 (0.882–0.977)	**0.982 (0.956–0.992)**	2/5
Forehand strike	0.818 (0.582–0.920)	0.875 (0.720–0.945)	0.891 (0.745–0.953)	0.955 (0.898–0.980)	0.936 (0.856–0.972)	2/5
Two-hand catch	**0.983 (0.961–0.992)**	0.899 (0.770–0.955)	0.908 (0.791–0.959)	**1.000**	**1.000**	5/5
Underhand throw	0.916 (0.809–0.963)	0.698 (0.333–0.865)	**0.966 (0.923–0.985)**	**0.958 (0.901–0.982)**	0.821 (0.597–0.921)	3/5
Ball Skills Subscale score	**0.970 (0.934–0.987)**	0.879 (0.689–0.949)	0.949 (0.884–0.978)	**0.967 (0.915–0.986)**	**0.962 (0.915–0.983)**	4/5
**Overall score**	0.953 (0.895–0.979)	0.907 (0.716–0.963)	**0.968 (0.927–0.986)**	**0.982 (0.941–0.993)**	**0.961 (0.912–0.983)**	4/5

* ICC: single-measurement, absolute-agreement, 2-way mixed-effects model, 95% confidence interval; **†**: Rater D (ER) coded slide skill with max punctuations in both test (live and slow-motion); In grey, skills with good-to-excellent intra-rater reliability (ICC & lower bound ≥ 0.75); in grey and bold, skills with excellent intra-rater reliability (ICC & lower bound ≥ 0.9).

**Table 2:** Descriptive statistics of overall score and locomotor and ball skills subscales’ scores. Results expressed as mean (standard deviation).

Raters		Locomotor Subscale		Ball Skills Subscale		Overall	
		Live	S-M	Live	S-M	Live	S-M
NR	A	34.2 (3.4)	33.2 (3.1)	36.0 (5.3)	36.4 (4.9)	70.2 (7.7)	69.5 (6.3)
	B	34.8 (4.3)	33.9 (4.4)	35.2 (4.4)	33.8 (5.0)	70.0 (7.1)	67.6 (7.9)
NR-PE	C	32.5 (4.6)	32.3 (4.5)	33.9 (5.0)	34.8 (4.3)	66.4 (7.9)	67.2 (7.6)
ER	D	32.6 (4.0)	32.9 (4.0)	39.7 (5.6)	40.6 (5.5)	72.2 (8.6)	73.4 (8.4)
	E	34.5 (3.2)	33.8 (3.6)	37.6 (5.0)	37.6 (4.4)	72.2 (6.4)	71.5 (6.3)

NR: Novice raters with no Physical Education background (A and B); NR-PE: Novice rater with Physical Education background (C); ER: Expert raters (D and E).

**Table 3:** Inter-rater reliability * among raters. Locomotor skills.

Raters	Test	Run	Slide †	Gallop	Hop	Horizontal Jump	Skip	ICC ≥ 0.5
Rater AxB	Live	0.074 (−0.323–0.450)	0.739 (0.492–0.876)	**0.768 (0.541–0.891)**	0.525 (0.171–0.758)	0.624 (0.311–0.815)	**0.856 (0.701–0.934)**	5/6
	S-M	−0.002 (−0.390–0.386)	**0.815 (0.624–0.914)**	**0.747 (0.505–0.880)**	0.581 (0.249–0.791)	0.497 (0.135–0.742)	**0.747 (0.506–0.880)**	4/6
Rater AxC	Live	0.106 (−0.294–0.475)	**0.791 (0.581–0.902)**	0.610 (0.290–0.807)	0.477 (0.109–0.730)	0.586 (0.255–0.793)	**0.939 (0.866–0.973)**	4/6
	S-M	0.090 (−0.309–0.462)	0.437 (0.058–0.705)	0.687 (0.407–0.849)	0.251 (−0.152–0.583)	0.688 (0.409–0.849)	**0.805 (0.607–0.909)**	3/6
Rater AxD	Live	0.129 (−0.273–0.493)	-----	**0.801 (0.600–0.907)**	0.496 (0.134–0.742)	0.626 (0.314–0.816)	0.612 (0.294–0.808)	3/5
	S-M	−0.052 (−0.431–0.643)	-----	**0.893 (0.773–0.952)**	0.385 (−0.004–0.672)	0.598 (0.274–0.801)	0.588 (0.259–0.795)	3/5
Rater AxE	Live	−0.022 (0.407–0.369)	0.647 (0.345–0.827)	0.672 (0.383–0.841)	0.297 (−0.103–0.614)	0.364 (−0.028–0.659)	0.710 (0.444–0.861)	2/6
	S-M	−0.106 (−0.474–0.295)	**0.749 (0.509–0.881)**	**0.771 (0.546–0.892)**	0.470 (0.100–0.726)	0.456 (0.082–0.717)	0.735 (0.485–0.874)	3/6
Rater BxC	Live	0.602 (0.279–0.803)	0.722 (0.464–0.867)	**0.883 (0.753–0.947)**	0.393 (0.005–0.678)	0.669 (0.379–0.839)	**0.760 (0.527–0.887)**	5/6
	S-M	0.491 (0.127–0.739)	0.451 (0.076–0.714)	0.723 (0.465–0.868)	0.423 (0.041–0.697)	0.597 (0.272–0.800)	**0.773 (0.550–0.893)**	3/6
Rater BxD	Live	0.471 (0.101–0.726)	-----	**0.874 (0.736–0.943)**	0.563 (0.224–0.781)	0.662 (0.369–0.836)	0.481 (0.114–0.732)	3/5
	S-M	0.629 (0.318–0.817)	-----	**0.814 (0.623–0.914)**	0.447 (0.072–0.712)	0.597 (0.272–0.800)	0.420 (0.038–0.695)	3/5
Rater BxE	Live	0.568 (0.231–0.784)	0.529 (0.177–0.761)	**0.816 (0.626–0.915)**	0.350 (−0.044–0.650)	0.492 (0.129–0.739)	0.732 (0.481–0.872)	3/6
	S-M	0.681 (0.399–0.846)	**0.759 (0.526–0.886)**	**0.808 (0.611–0.910)**	0.692 (0.415–0.851)	0.585 (0.255–0.793)	0.608 (0.288–0.806)	6/6
Rater CxD	Live	0.569 (0.232–0.784)	-----	**0.827 (0.646–0.920)**	0.656 (0.356–0.832)	**0.784 (0.568–0.899)**	0.636 (0.330–0.822)	5/5
	S-M	0.528 (0.176–0.760)	-----	**0.766 (0.538–0.890)**	0.552 (0.209–0.774)	**0.767 (0.539–0.890)**	0.652 (0.353–0.830)	5/5
Rater CxE	Live	0.587 (0.258–0.794)	0.483 (0.117–0.734)	**0.841 (0.672–0.927)**	0.457 (0.084–0.718)	0.407 (0.023–0.687)	0.702 (0.432–0.857)	3/6
	S-M	0.683 (0.401–0.847)	0.640 (0.336–0.824)	0.704 (0.434–0.858)	0.423 (0.042–0.697)	0.591 (0.263–0.796)	0.655 (0.358–0.832)	5/6
Rater DxE	Live	0.551 (0.207–0.774)	-----	**0.766 (0.538–0.890)**	0.183 (−0.221–0.533)	0.637 (0.330–0.822)	0.483 (0.117–0.734)	3/5
	S-M	0.459 (0.086–0.719)	-----	**0.826 (0.645–0.920)**	0.433 (0.054–0.703)	0.658 (0.362–0.833)	0.591 (0.263–0.796)	3/5
All raters	Live	0.423 (0.243–0.629)	0.494 (0.314–0.686)	**0.795 (0.674–0.889)**	0.431 (0.251–0.636)	0.597 (0.425–0.762)	**0.693 (0.540–0.827)**	3/6
	S-M	0.368 (0.192–0.581)	0.499 (0.319–0.690)	**0.770 (0.641–0.875)**	0.468 (0.287–0.666)	0.616 (0.446–0.775)	0.652 (0.489–0.800)	3/6

Rater A and B: Novice raters with no Physical Education background; Rater C: Novice rater with Physical Education background; Rater D & E: Expert raters. * ICC: single-measurement, consistency, 2-way random-effects model, 95% confidence interval; † Rater D (ER) coded slide skill with max punctuations in both test (live and slow-motion); In grey, skills with ICC ≥ 0.5; in grey and bold, skills with at least moderate inter-rater reliability (ICC & lower bound ≥ 0.5).

**Table 4:** Inter-rater reliability * among raters. Ball skills.

Raters	Test	Two-Hand Strike	One-Hand Stationary Dribble	Overhand Throw	Kick	Forehand Strike	Two-Hand Catch	Underhand Throw	ICC ≥ 0.5
Rater AxB	Live	**0.779 (0.561–0.896)**	0.372 (−0.019–0.664)	0.548 (0.204–0.772)	0.393 (0.006–0.678)	0.632 (0.323–0.819)	0.669 (0.379–0.839)	0.348 (−0.046–0.649)	4/7
	S-M	**0.751 (0.512–0.882)**	**0.778 (0.559–0.896)**	0.695 (0.420–0.853)	0.289 (−0.112–0.609)	0.639 (0.333–0.823)	0.621 (0.306–0.813)	0.598 (0.272–0.800)	6/7
Rater AxC	Live	**0.805 (0.607–0.909)**	0.396 (0.009–0.680)	**0.783 (0.567–0.898)**	0.579 (0.246–0.790)	0.569 (0.232–0.784)	**0.876 (0.739–0.943)**	0.589 (0.261–0.796)	6/7
	S-M	**0.788 (0.576–0.901)**	0.631 (0.322–0.819)	0.660 (0.365–0.834)	0.478 (0.110–0.730)	0.674 (0.387–0.842)	**0.929 (0.845–0.968)**	0.475 (0.106–0.729)	5/7
Rater AxD	Live	**0.754 (0.517–0.883)**	0.736 (0.488–0.875)	0.586 (0.255–0.793)	0.367 (−0.025–0.661)	0.522 (0.167–0.757)	0.392 (0.005–0.677)	0.594 (0.267–0.798)	5/7
	S-M	0.630 (0.320–0.818)	0.698 (0.426–0.855)	0.445 (0.069–0.710)	0.532 (0.181–0.763)	0.549 (0.204–0.772)	0.389 (0.001–0.675)	0.526 (0.174–0.759)	5/7
Rater AxE	Live	0.660 (0.366–0.835)	0.560 (0.219–0.779)	0.570 (0.233–0.785)	0.500 (0.139–0.744)	0.080 (−0.318–0.454)	0.308 (−0.091–0.622)	0.264 (−0.138–0.592)	4/7
	S-M	0.732 (0.480–0.872)	0.545 (0.199–0.770)	0.476 (0.107–0.729)	0.412 (0.028–0.690)	0.378 (−0.012–0.668)	0.297 (−0.103–0.614)	0.322 (−0.076–0.631)	3/7
Rater BxC	Live	0.698 (0.426–0.855)	**0.900 (0.787–0.955)**	**0.749 (0.508–0.881)**	0.404 (0.019–0.685)	0.647 (0.346–0.828)	0.600 (0.276–0.801)	0.469 (0.099–0.725)	5/7
	S-M	0.681 (0.398–0.846)	0.735 (0.486–0.874)	**0.776 (0.554–0.895)**	0.462 (0.090–0.721)	0.470 (0.100–0.726)	0.705 (0.436–0.858)	0.614 (0.297–0.810)	5/7
Rater BxD	Live	0.544 (0.198–0.770)	0.337 (−0.058–0.642)	0.462 (0.090–0.721)	0.740 (0.493–0.876)	0.691 (0.414–0.851)	0.308 (−0.091–0.622)	0.540 (0.192–0.767)	4/7
	S-M	0.582 (0.250–0.791)	0.643 (0.340–0.825)	0.373 (−0.018–0.665)	**0.778 (0.559–0.896)**	0.611 (0.292–0.808)	0.228 (−0.176–0.566)	0.636 (0.330–0.822)	5/7
Rater BxE	Live	0.652 (0.354–0.830)	0.385 (−0.004–0.673)	0.596 (0.271–0.800)	0.533 (0.182–0.763)	0.361 (−0.032–0.657)	0.302 (−0.097–0.618)	0.292 (−0.109–0.611)	3/7
	S-M	**0.809 (0.614–0.911)**	0.580 (0.248–0.790)	0.608 (0.288–0.806)	0.534 (0.184–0.764)	0.482 (0.116–0.733)	0.227 (−0.177–0.565)	0.285 (−0.117–0.606)	4/7
Rater CxD	Live	0.716 (0.453–0.864)	0.426 (0.045–0.699)	0.610 (0.290–0.807)	0.389 (0.001–0.675)	0.600 (0.276–0.802)	0.410 (0.026–0.689)	0.711 (0.447–0.892)	4/7
	S-M	0.487 (0.121–0.736)	0.593 (0.267–0.798)	0.684 (0.402–0.847)	0.339 (−0.057–0.642)	0.565 (0.226–0.782)	0.313 (−0.086–0.625)	0.665 (0.374–0.837)	4/7
Rater CxE	Live	0.682 (0.400–0.846)	0.491 (0.127–0.739)	0.589 (0.261–0.796)	0.412 (0.028–0.690)	0.357 (−0.036–0.655)	0.303 (−0.097–0.618)	0.248 (−0.155–0.580)	2/7
	S-M	0.615 (0.298–0.810)	0.477 (0.109–0.730)	0.570 (0.234–0.785)	0.378 (−0.012–0.668)	0.409 (0.024–0.688)	0.222 (−0.181–0.562)	0.377 (−0.014–0.667)	2/7
Rater DxE	Live	0.574 (0.239–0.787)	0.581 (0.249–0.791)	0.505 (0.146–0.747)	0.600 (0.277–0.802)	0.241 (−0.162–0.575)	0.586 (0.255–0.793)	0.176 (−0.228–0.528)	5/7
	S-M	0.530 (0.187–0.762)	0.525 (0.172–0.759)	0.343 (−0.052–0.645)	0.615 (0.297–0.810)	0.287 (−0.114–0.607)	0.586 (0.255–0.793)	0.466 (0.095–0.724)	3/7
All raters	Live	**0.686 (0.531–0.823)**	0.518 (0.339–0.705)	0.595 (0.423–0.761)	0.500 (0.320–0.691)	0.470 (0.289–0.667)	0.496 (0.316–0.688)	0.452 (0.271–0.653)	4/7
	S-M	0.652 (0.489–0.800)	0.627 (0.459–0.783)	0.558 (0.381–0.734)	0.498 (0.318–0.689)	0.502 (0.322–0.692)	0.496 (0.316–0.688)	0.506 (0.326–0.695)	5/7

Rater A and B: Novice raters; Rater C: Novice rater with Physical Education background; Rater D & E: Expert raters. * ICC: single-measurement, consistency, 2-way random-effects model, 95% confidence interval; In grey, skills with ICC ≥ 0.5; in grey and bold, skills with at least moderate inter-rater reliability (ICC & lower bound ≥ 0.5).

## Data Availability

No new data were created or analyzed in this study. Data sharing is not applicable to this article.

## References

[1] Gallahue D.L., Donnelly F.C. (2007). Developmental Physical Education for All Children.

[2] Gallahue D., Ozmun J., Goodway J. (2012). Understanding Motor Development: Infants, Children, Adolescents, Adults.

[3] Logan S.W., Ross S.M., Chee K., Stodden D.F., Robinson L.E. (2018). Fundamental motor skills: A systematic review of terminology. J. Sports Sci..

[4] Lubans D.R., Morgan P.J., Cliff D.P., Barnett L.M., Okely A.D. (2010). Fundamental Movement Skills in Children and Adolescents. Review of associated health benefits. Sports Med..

[5] Cattuzzo M.T., Henrique R.D.S., Ré A.H.N., De Oliveira I.S., Melo B.M., Moura M.D.S., De Araújo R.C., Stodden D. (2016). Motor competence and health related physical fitness in youth: A systematic review. J. Sci. Med. Sport.

[6] Barnett L.M., Lai S.K., Veldman S.L.C., Hardy L.L., Cliff D.P., Morgan P.J., Zask A., Lubans D.R., Shultz S.P., Ridgers N.D. (2016). Correlates of Gross Motor Competence in Children and Adolescents: A Systematic Review and Meta-Analysis. Sports Med..

[7] Robinson L.E., Stodden D.F., Barnett L.M., Lopes V.P., Logan S.W., Rodrigues L.P., D’Hondt E. (2015). Motor Competence and its Effect on Positive Developmental Trajectories of Health. Sports Med..

[8] Haapala E.A. (2013). Cardiorespiratory Fitness and Motor Skills in Relation to Cognition and Academic Performance in Children—A Review. J. Hum. Kinet..

[9] Costa H.J.T., Barcala-Furelos R., Abelairas-Gómez C., Arufe-Giráldez V. (2015). The Influence of a Structured Physical Education Plan on Preschool Children’s Psychomotor Development Profiles. Australas. J. Early Child..

[10] Rintala P., Sääkslahti A., Iivonen S. (2017). Reliability Assessment of Scores from Video-Recorded TGMD-3 Performances. J. Mot. Learn. Dev..

[11] Logan S.W., Robinson L.E., Rudisill M.E., Wadsworth D.D., Morera M. (2014). The comparison of school-age children’s performance on two motor assessments: The Test of Gross Motor Development and the Movement Assessment Battery for Children. Phys. Educ. Sport Pedagog..

[12] Piek J.P., Dawson L., Smith L.M., Gasson N. (2008). The role of early fine and gross motor development on later motor and cognitive ability. Hum. Mov. Sci..

[13] Skinner R.A., Piek J.P. (2001). Psychosocial implications of poor motor coordination in children and adolescents. Hum. Mov. Sci..

[14] Burton A., Miller D. (1998). Movement Skill Assessment.

[15] Yun J., Shapiro D.R. (2004). A Quantitative Approach to Movement Skill Assessment for Children with Mental Retardation. Adapt. Phys. Act. Q..

[16] Clark J.E., Ramachandran V.S. (1994). Motor Development. Encyclopedia of Human Behavior.

[17] Payne V.G., Isaacs L.D. (2008). Human Motor Development.

[18] Ulrich D.A. (2000). Test of Gross Motor Development.

[19] Ulrich D.A. (2019). Test of Gross Motor Development.

[20] Rey E., Carballo-Fazanes A., Varela-Casal C., Abelairas-Gómez C. (2020). Reliability of the test of gross motor development: A systematic review. PLoS ONE.

[21] Akuffo P.B., Hodge S.R. (2008). Roles and Responsibilities of Adapted Physical Education Teachers in an Urban School District. Educ. Urban Soc..

[22] Palmer K.K., Brian A. (2016). Test of Gross Motor Development-2 Scores Differ Between Expert and Novice Coders. J. Mot. Learn. Dev..

[23] Koo T.K., Li M.Y. (2016). A Guideline of Selecting and Reporting Intraclass Correlation Coefficients for Reliability Research. J. Chiropr. Med..

[24] Aye T., Oo K.S., Khin M.T., Kuramoto-Ahuja T., Maruyama H. (2017). Reliability of the test of gross motor development second edition (TGMD-2) for Kindergarten children in Myanmar. J. Phys. Ther. Sci..

[25] Capio C.M., Eguia K.F., Simons J. (2015). Test of gross motor development-2 for Filipino children with intellectual disability: Validity and reliability. J. Sports Sci..

[26] Farrokhi A., Zareh Z., Karimi A., Kazemnejad A., Ilbeigi S. (2014). Reliability and validity of test of gross motor development-2 (Ulrich, 2000) among 3–10 aged children of Tehran City. J. Phys. Educ. Sport Manag..

[27] Houwen S., Hartman E., Jonker L., Visscher C. (2010). Reliability and Validity of the TGMD-2 in Primary-School-Age Children with Visual Impairments. Adapt. Phys. Act. Q..

[28] Allen K.A., Bredero B., Van Damme T., Ulrich D.A., Simons J. (2017). Test of Gross Motor Development-3 (TGMD-3) with the Use of Visual Supports for Children with Autism Spectrum Disorder: Validity and Reliability. J. Autism Dev. Disord..

[29] Estevan I., Molina-García J., Queralt A., Álvarez O., Castillo I., Barnett L. (2017). Validity and Reliability of the Spanish Version of the Test of Gross Motor Development–3. J. Mot. Learn. Dev..

[30] Mohammadi F., Bahram A., Khalaji H., Ulrich D., Ghadiri F. (2019). Evaluation of the Psychometric Properties of the Persian Version of the Test of Gross Motor Development–3rd Edition. J. Mot. Learn. Dev..

[31] Maeng H., Webster E.K., Pitchford E., Ulrich D. (2017). Inter- and Intrarater Reliabilities of the Test of Gross Motor Development—Third Edition among Experienced TGMD-2 Raters. Adapt. Phys. Act. Q..

[32] Valentini N.C., Zanella L.W., Webster E.K. (2017). Test of Gross Motor Development—Third Edition: Establishing Content and Construct Validity for Brazilian Children. J. Mot. Learn. Dev..

[33] Wagner M.O., Webster E.K., Ulrich D.A. (2017). Psychometric Properties of the Test of Gross Motor Development, Third Edition (German Translation): Results of a Pilot Study. J. Mot. Learn. Dev..

[34] Kim Y., Park I., Kang M. (2012). Examining Rater Effects of the TGMD-2 on Children with Intellectual Disability. Adapt. Phys. Act. Q..

[35] Ayán C., Cancela J., Sanchez-Lastra M.A., Carballo-Roales A., Domínguez-Meis F., Redondo-Gutiérrez L. (2019). Fiabilidad y Validez de la Batería TGMD-2 en Población Española. Rev. Iberoam. Diagnóstico Eval..

[36] Barnett L.M., Minto C., Lander N., Hardy L.L. (2014). Interrater reliability assessment using the Test of Gross Motor Development-2. J. Sci. Med. Sport.

[37] Onate J., Cortes N., Welch C., Van Lunen B.L. (2010). Expert Versus Novice Interrater Reliability and Criterion Validity of the Landing Error Scoring System. J. Sport Rehabil..

[38] Minick K.I., Kiesel K.B., Burton L., Taylor A., Plisky P., Butler R.J. (2010). Interrater Reliability of the Functional Movement Screen. J. Strength Cond. Res..

[39] Fleiss J.L. (1986). Design and Analysis of Clinical Experiments.

[40] Cicchetti D.V. (1994). Guidelines, criteria, and rules of thumb for evaluating normed and standardized assessment instruments in psychology. Psychol. Assess..

